# Antioxidant and Antimicrobial Activity of *Alpinia officinarum*

**DOI:** 10.4103/0250-474X.62233

**Published:** 2010

**Authors:** A. R. Srividya, S. P. Dhanabal, V. K. Misra, G. Suja

**Affiliations:** Department of Pharmaceutical Biotechnology, J. S. S. College of Pharmacy, Rocklands, Ooty-643 001, India

**Keywords:** *Alpinia officinarum*, antimicrobial, antioxidant, cold and hot maceration, free radical scavenging, hydroalcoholic extract

## Abstract

*Alpinia officinarum* is a rhizome belonging to the family zingeberaeceae. Hydro alcoholic extract by hot and cold maceration and methanol extract by percolation process Qualitative phytochemical analysis of extract of *Alpinia officinarum* rhizome showed a majority of the compound including tannins, alkaloids, flavonoids and saponins. Hydroalcoholic extract prepared by hot maceration process was found to contain more phenol and flavonol and it was measured as 50.1 mg/g and 54.02 mg/g, respectively. All the three extracts showed moderate to potent antimicrobial activity against the *Bacillus cereus, Staphylococcus aureas, Pseudomonas auroginosa, Escherichia coli*. None of the extracts showed antifungal activity against *Aspergillus niger* and *Candida albicans*. All the three extracts showed a concentration dependent radical scavenging activity by inhibiting diphenylpicrylhydrazyl free radical at the same time hydroalcoholic extract prepared by hot maceration process showed better reducing and total antioxidant activity.

This paper deals with the antioxidant and antimicrobial activity of *Alpinia officinarum* which is not scientifically proven. *Alpinia officinarum* is a rhizome belonging to the family, zingeberaeceae, cultivated in South East Asia. This rhizome is characterized by dark reddish brown colour which has a strong aromatic odour. Ethnomedical uses for this rhizome is found to be against rheumatism, bronchial catarrh, bad breath, ulcers, whooping cough in children, throat infections to control incontinence[1–3]. Plant material was collected in the month of May 2007 from the local market in Ooty and authenticated at the Medicinal Survey and Collection Unit, Government Arts College, Ootacamund, Tamil Nadu, India. The dried rhizomes of *Alpinia officinarum* were powdered and extracted with 50 % ethanol by hot and cold maceration. The extract was filtered and the filtrate was evaporated to dryness in a rotary evaporator to yield dark brown semi-solids. The powdered rhizome was extracted with methanol by percolation method. The extract yielded a dark brown semi solid. All the extracts were stored in the refrigerator till use[4].

The extracts obtained were tested for the qualitative chemical tests for the identification of various phytoconstituents such as alkaloids, carbohydrates, protein, amino acids, steroids and sterols, glycosides, flavonoids, tannins, triterpinoids, fixed oil[5,6]. Total phenol content of the extract was determined by using Folin-Ciocalteu method[7]. This test is based on the oxidation of phenolic groups with phosphomolybdic and phosphotungstic acids. After oxidation a green-blue complex formed is measured at 750 nm. In a series of test tubs, 0-4 ml of the extract (1 mg/ml to 0.1 mg/ml) in methanol was taken, mixed with 2 ml of Folin-Ciocalteu reagent in distilled water (1: 10) and 1.6 ml of sodium carbonate. After shaking, it was kept for 2 h for the reaction to take place. Then absorbance was measured at 750 nm. Using gallic acid monohydrate, standard curve was prepared and the linearity was obtained in the range of 1-10 μg/ml. Using the standard curve, the total phenol content was obtained. The total phenol content was expressed as gallic acid equivalent in mg/g of the extract[7]. The extract 0.5 ml was separately mixed with 1.5 ml of methanol, 0.1 ml of 10% AlCl_3_, 0.1 ml of 1 M potassium acetate and 2.8 ml of distilled water. After incubation at 30°, the absorbance of the reaction mixture was measured at 415 nm. Using rutin, standard curve was prepared and linearity was obtained in the range of 1-10 μg/ml. Using the standard curve total flavonol content was expressed as rutin equivalent, in mg/g or percentage w/w of the extract[8].

Evaluation of antimicrobial activity was performed by Cup plate method. Sterile nutrient agar/Sabouraud dextrose agar plates were prepared by pouring the sterilized media in sterile Petri plates under aseptic conditions. The test organism 0.1 ml was spread on agar plates. Cups were made at the size of 5 mm diameter, in the agar plates using the sterile borer. Drug as well as the standard and DMSO solvent control were added into the pores separately. The plates were maintained at +4° for 1 h to allow the diffusion of solution into the medium. All the plates containing bacteria were incubated at 37° for 24 h and that of fungi at 28° for 48 h[9].

*In vitro* antioxidant activity was evaluated by diphenyl picryl hydrazyl (DPPH) radical scavenging method. The principle of this assay is based on the measurement of the scavenging ability of the antioxidant towards the stable radical. The free radical DPPH is reduced to the corresponding hydrazine when it reacts with hydrogen donors, this stability is evaluated by the decolouration assay which evaluates the decrease in absorbance at 518-528 nm produced by the addition of the antioxidant to a DPPH solution in ethanol or methanol. The assay was carried out in a 96 well microtitre plate. To 200 μl of DPPH solution, 10 μl of each of the test sample or the standard solution were added separately in the wells of the microtitre plate. The final concentration of the test and the standard solution used were 1000 μl to 0.9765 μl. The plates were incubated at 37° for 20 min and the absorbance of each solution was measured at 490 nm using ELISA reader against the corresponding test and standard and the remaining DPPH was calculated. IC_50_ is the concentration of the sample required to scavenge 50% of DPPH free radical[10]. Percentage of inhibition is calculated by subtracting the absorbance of the sample from the absorbance of the control divided by absorbance of the control.

The reducing power of the extract was determined according to the method of Oyaizu[11]. Different concentrations of the extract (100-1000 μl) in 1 ml of distilled water were mixed with phosphate buffer and potassium ferricyanide. The mixture was incubated at 50° for 20 min. A portion (2.5 ml) of trichloroacetic acid (10%) was added to the mixture, which was then centrifuged at 3,000 rpm for 10 min. The supernatant solution (2.5 ml) was mixed with distilled water (2.5 ml) and ferric chloride (0.5 ml of 0.1%) and the absorbance was measured at 700 nm. Increased absorbance of the reaction mixture indicated increased reducing power. Ascorbic acid was used as a reference standard; phosphate buffer (PH 6.6) was used as blank solution[11]. The absorbance of the final reaction mixture of two parallel experiments were taken was expressed as mean±standard deviation. Total antioxidant activity of the extract was evaluated by the phosphomolybdenum method. The assay is based on the reduction of Mo (V1) to Mo (V) by the extract and the subsequent formation of a green phosphate/Mo(V) complex at acidic pH.

The extract 0.1 ml was combined with 3 ml of the reagent solution (0.6 M sulphuric acid, 28 mM sodium phosphate and 4 mM ammonium molbdate). The tubes containing the reaction solution were incubated at 95° for 90 min. The absorbance of the solution was measured at 695 nm using spectrophotometer against blank after cooling to room temperature. Methanol (0.3 ml) in the place of extract was used as the blank. The antioxidant activity is expressed as the number of equivalent of ascorbic acid[11,12].

Qualitative phytochemical analysis of extract of *Alpinia officinarum* rhizome showed a majority of the compound including tannins, alkaloids, flavonoids and saponins and the results are tabulated in the Table 1. The total phenol content of the extracts of *Alpinia officinarum* rhizome is expressed as gallic acid equivalent and the total flavonol content of the extract is expressed as rutin equivalent and hydroalcoholic extract prepared by hot maceration process was found to contain more phenol and flavonol and it was measured as 50.1 and 54.02 mg/g, respectively. The results for the total phenol and favonol were tabulated in the Table 2. All the extracts at 1000 μg/ml showed significant anti bacterial activity in comparison with the standard but none of the extracts showed antifungal activity even at higher concentrations. The results for the antimicrobial activity are tabulated in the Table 3. All the three extracts showed moderate to potent antibacterial activity against the *B. cereus, Staph. aureas, P. aeruginosa* and *E. coli*. None of the extracts showed antifungal activity against *Aspergillus niger* and *Candida albicans*. The hydroalcoholic extract prepared by hot maceration showed minimum inhibitory concentration in the range of 31.25 to 500 μg/ml. The results for the minimum inhibitory concentration were tabulated in Table 4. The hydroalcoholic extract prepared by hot maceration process showed a concentration-dependent radical scavenging activity by inhibiting DPPH free radical. All the three extracts showed moderate reducing power when compared to ascorbic acid which has taken as the standard antioxidant. Hydroalcoholic extract prepared by hot maceration showed better antioxidant activity than the other extracts. The results for the antioxidant activity were tabulated in the Table 5.

**TABLE 1:** QUALITATIVE PHYTOCHEMICAL ANALYSIS OF *ALPINIA OFFICINARUM* RHIZOME

Constituents	HA(HM)	HA(CM)	ME(PER)
Alkaloids	+	+	+
Glycosides	-	-	-
Terpenoids	+	+	-
Saponins	+	+	+
Tannins	+	+	+
Protein and amino acids	+	+	+
Flavonoids	+	+	+
Steroids	+	+	-

HA(HM) is hydroalcoholic extract prepared by hot maceration process, HA(CM) is hydroalcoholic extract prepared by cold maceration process and ME(PER) represents methanol extract prepared by percolation process.

+ indicates the presence

- indicates the absence.

**TABLE 2:** QUANTITATIVE PHYTOCHEMICAL CONSTITUENTS ANALYSIS OF *ALPINIA OFFICINARUM*

Name of the extract	Total phenol content (mg/g)	Total flavonol content (mg/g)
HA(HM)	50.1	54.02
HA(CM)	41.35	36.36
ME(PER)	30.6	27.64

HA(HM) is hydroalcoholic extract prepared by hot maceration process, HA(CM) is hydroalcoholic extract prepared by cold maceration process and ME(PER) represents methanol extract prepared by percolation process.

**TABLE 3:** ANTIMICROBIAL ACTIVITY OF *ALPINIA OFFICINARUM* RHIZOME

Microorganism	Zone of Inhibition (mm)										
	
	HA(HM)			HA(CM)			ME(PER)			Standard
			
	1000 μg	500μg	250μg	1000μg	500μg	250 μg	1000μg	500 μg	250 μg	
Gram Positive										Tetracycline 25
*Bacillus cereus*	27	23	18	22	17	13	19	17	12	25
*Staphylococcus aureus*	38	29	24	29	27	26	27	26	18	35
Gram negative										
*Pseudomonas aeruginosa*	17	15	14	16	14	13	12	12	09	20
Escherichia coli	23	18	13	17	16	11	09	07	04	19
Fungi										Amphotericin B
*Candida albicans*	08	06	05	06	00	00	00	00	00	11
Aspergillus niger	06	03	00	04	00	00	00	00	00	08

HA(HM) is hydroalcoholic extract prepared by hot maceration process, HA(CM) is hydroalcoholic extract prepared by cold maceration process and ME(PER) represents methanol extract prepared by percolation process. Vehicle control containing DMSO did not produce any inhibition

**TABLE 4:** MINIMUM INHIBITORY CONCENTRATION OF *ALPINIA OFFICINARUM* RHIZOME

Microorganisms	MIC of Rhizome extract (μg/ml)		
	
	HA (HM)	HA(CM)	ME(PER)
Gram Positive			
*Bacillus cereus*	250	500	250
*Staphylococcus aureus*	31.25	125	250
Gram negative			
*Pseudomonas auroginosa*	250	500	>1000
*Escherichia coli*	250	500	>1000
Fungi			
*Candida albicans*	500	1000	>1000
*Aspergillus niger*	500	500	>1000

HA(HM) is hydroalcoholic extract prepared by hot maceration process, HA(CM) is hydroalcoholic extract prepared by cold maceration process and ME(PER) represents methanol extract prepared by percolation process. MIC is Minimum inhibitory concentration expressed in μg/ml

**TABLE 5:** ANTIOXIDANT ACTIVITY OF *ALPINIA OFFICINARUM* RHIZOME

Name of the Extract	IC_50_ Value of DPPH (μg/ml)	Reducing power	Total antioxidant activity nM equivalent of ascorbic acid
HA (HM)	95.41±1.10	60.71±0.36	2.868±0.044
HA(CM)	123.43±0.78	59.8±0.05	2.528±0.025
ME(PER)	137.33±1.10	59.70±0.05	2.542±0.116
Ascorbic acid	2.75±0.09	3.50±0.05	-

HA(HM) is hydroalcoholic extract prepared by hot maceration process, HA(CM) is hydroalcoholic extract prepared by cold maceration process and ME(PER) represents methanol extract prepared by percolation process.
